# Occludin is overexpressed in tubo-ovarian high-grade serous carcinoma compared to mesothelioma and is a marker of tumor progression and chemoresistance

**DOI:** 10.1007/s10585-023-10251-5

**Published:** 2023-12-23

**Authors:** Margarida Varela dos Santos, Arild Holth, Katharina Bischof, Ben Davidson

**Affiliations:** 1 Department of Pathology, Oslo University Hospital, Norwegian Radium Hospital, N-0310 Montebello, Oslo, Norway; 2 Permanent Address: Serviço de Anatomia Patológica, Centro Hospitalar Universitário de Lisboa Central E.P.E, Rua José António Serrano, 1150-199 Lisbon, Portugal; 3 Department of Gynecologic Oncology, Oslo University Hospital, Norwegian Radium Hospital, N-0310 Oslo, Norway; 4 Faculty of Medicine, Institute of Clinical Medicine, University of Oslo, N-0316 Oslo, Norway

**Keywords:** Occludin, Immunohistochemistry, High-grade serous carcinoma, Mesothelioma, Effusion

## Abstract

The objective of this study was to analyze the expression and prognostic role of the tight junction protein occludin in high-grade serous carcinoma (HGSC). Occludin protein expression by immunohistochemistry was analyzed in 602 HGSC (417 effusions, 185 surgical specimens). Expression in mesothelioma (n = 87; 45 effusions, 42 surgical specimens) was studied for comparative purposes. Occludin protein expression was found in 587/602 (98%) HGSC vs. 40/87 (46%) mesotheliomas and was predominantly limited to < 5% of cells in the latter (p < 0.001). Occludin was additionally overexpressed in HGSC effusions compared to surgical specimens (p < 0.001) and was overexpressed in post-chemotherapy effusions compared to chemo-naive effusions tapped at diagnosis (p = 0.015). Occludin expression in HGSC surgical specimens was associated with poor chemoresponse (p < 0.001) and primary resistance (p = 0.001). Expression in effusions and surgical specimens was unrelated to survival (p > 0.05). In conclusion, occludin expression is higher in HGSC compared to mesothelioma, and this protein is overexpressed in HGSC effusions, possibly reflecting changes in adhesion related to anchorage-independent growth in this microenvironment. Overexpression in post-chemotherapy compared to chemo-naïve effusions suggest a role in disease progression. Occludin expression in surgical specimens may be related to chemoresistance.

**Supplementary Information:**

The online version contains supplementary material available at 10.1007/s10585-023-10251-5.

## Introduction

Ovarian cancer is the 8th most common and the 8th most lethal cancer in women, with 313,959 new diagnoses and 207,252 deaths registered in 2020 globally [1]. More than 90% of ovarian cancers are tubo-ovarian carcinomas, and 70% of the latter are high-grade serous carcinomas (HGSC), of which the majority have origin in the fallopian tube, mainly its fimbrial region [2].

Tubo-ovarian carcinoma may be clinically indistinguishable from mesothelioma, primary tumor of the serosal cavities, particularly when carcinomatosis is present at advanced stage. Globally, 30,870 patients were diagnosed with mesothelioma, and 26,278 died of this cancer in 2020, of whom 9310 and 7597 were females, respectively [1]. While more common in the pleural cavity, this disease also affects the peritoneal cavity, where incidence in women is proportionally higher than in the pleural cavity.

Peritoneal dissemination of HGSC, as well as mesothelioma, reduces the possibility to cure patients diagnosed with these malignancies, and necessitates combination therapy consisting of debulking surgery and chemotherapy, as well as targeted therapy. In view of the fact that the majority of HGSC patients eventually succumb to their disease, identification of biomarkers related to disease progression in metastatic disease is important.

Epithelial cells form the first line of defense in mucosal membranes. Tight junctions, which seal the paracellular space, are critical in this respect. Proteins that form the tight junction protein complex belong to the claudin, zonula occludens and tight junction-associated MARVEL protein (TAMP) families. Occludin, member of the TAMP family, is a 522 amino acid transmembrane protein considered to be regulator, rather than essential structural component, of tight junctions [3]. Occludin plays a role in endocytosis and is co-receptor for Hepatitis C and Coxsackie virus [3]. Experimental data suggest that occludin may regulate epithelial cell apoptosis and survival [3].

Studies of occludin expression in tubo-ovarian carcinomas are to date limited to a single report in which expression of this protein was compared between 36 serous carcinomas and 26 serous cystadenomas, in which expression did not significantly differ between these two categories. No association was observed with clinical parameters [4]. The series analyzed was nevertheless small, and the fact that the study was performed prior to implementation of the 2014 WHO classification raises the possibility that it included both HGSC and low-grade serous carcinomas (LGSC).

The objective of the present study was to analyze the clinical role of occludin in a large series of well-characterized HGSC. In addition, the diagnostic role of this protein in the differential diagnosis from mesothelioma was investigated.

## Material and methods

### Patients and specimens

HGSC specimens consisted of 602 tumors (417 effusions, 185 surgical specimens).

The 417 effusions were from 417 patients and consisted of 361 peritoneal and 56 pleural specimens submitted to the Department of Pathology at the Norwegian Radium Hospital during the period of 1998 to 2017. Data regarding chemotherapy status were available for 408 of 417 patients with HGSC effusions. The majority of these (n = 272) were chemo-naïve, whereas a smaller group (n = 136) were post-chemotherapy specimens, the majority tapped at disease recurrence.

The 185 surgical specimens were from 114 patients (53 patients with 1 lesion, 52 with 2 lesions, 8 with 3 lesions, and 1 with tumors from 4 anatomic locations) and consisted of ovarian, peritoneal and omental tumors resected in the period 1996–2007. Thirty-one patients had matched effusion and one or more surgical specimens. As the SEE-FIM (Sectioning and Extensively Examining the Fimbria) procedure, required for assessing whether the tumor originates from the fallopian tube, has not been applied during this period, specimens are referred to as HGSC, without reference to primary vs. metastatic location, though the latter is assumed for the majority.

A series of pleural and peritoneal mesotheliomas (n = 87; 45 effusions, 42 surgical specimens) from patients diagnosed with epithelioid or biphasic mesothelioma, confirmed in biopsy specimens, was studied for comparative purposes. Specimens were submitted to the Department of Pathology at the Norwegian Radium Hospital during the period of 1986 to 2008.

Cell blocks from effusion pellets were prepared using the thrombin clot protocol. Effusions were diagnosed based on smear and cell block morphology and immunohistochemistry (IHC) by an experienced cytopathologist (BD) applying established guidelines [5]. Surgical specimens were similarly diagnosed based on morphology and IHC by an experienced gynecologic pathologist (BD) based on established guidelines [2].

Clinicopathologic data for the HGSC effusion and surgical specimen cohorts are detailed in Table 1 and 2, respectively.

**Table 1:** Clinicopathologic parameters of the HGSC effusion cohort (n = 417)

Parameter	Distribution
Age (mean)	23–88 years (62)
FIGO stage	
I	5
II	7
III	245
IV	153
NA	7
Residual disease	
0	73
≤ 1 cm	136
> 1 cm	119
NA	89
CA 125 at diagnosis (range; median)^*a*^	10–62400 (1200)
Primary treatment	
Debulking surgery	252
Neoadjuvant chemotherapy	120
Only chemotherapy	32
No treatment	2
NA	11
Chemoresponse after primary treatment	
CR	197
PR	102
SD	32
PD	36
NA^*b*^	50

*NA* not available; *CR* complete response; *PR* partial response; *SD* stable disease; *PD *progressive disease

^*a*^Available for 332 patients

^*b*^Not available (missing data or disease response after chemotherapy could not be evaluated because of normalized CA 125 after primary surgery or missing CA 125 information and no residual tumor)

**Table 2:** Clinicopathologic parameters of the HGSC surgical specimen cohort (n = 91 patients with data)

Parameter	Distribution
Age range (mean)	23–88 years (62)
FIGO stage	
III	61
IV	29
NA	1
Residual disease	
0	11
≤ 1 cm	28
> 1 cm	28
NA	24
CA 125 at diagnosis (range; median)^*a*^	51–40,719 (1371)
Primary treatment	
Debulking surgery	62
Neoadjuvant chemotherapy	28
NA	1
Chemoresponse after primary treatment	
CR	52
PR	17
SD	3
PD	10
NA^*b*^	9

*NA* not available; *CR* complete response; *PR* partial response; *SD* stable disease; *PD* progressive disease

^*a*^Available for 88 patients

^*b*^Not available (missing data or disease response after chemotherapy could not be evaluated because of normalized CA 125 after primary surgery or missing CA 125 information and no residual tumor)

Informed consent was obtained according to national and institutional guidelines. Study approval was given by the Regional Committee for Medical Research Ethics in Norway (S-04300).

### IHC

Formalin-fixed, paraffin-embedded sections from the above-described 689 tumors were analyzed for occludin protein expression using the Dako EnVision Flex + System (K8012; Dako, Glostrup, Denmark). The occludin antibody was a mouse monoclonal antibody purchased from Santa Cruz Biotechnology (cat # Sc-133255, clone F-11; Santa Cruz, CA), applied at a 1:50 dilution following antigen retrieval in HpH buffer (pH 9.0).

Following deparaffinization, sections were treated with EnVision™ Flex + mouse linker (15 min) and EnVision™ Flex/HRP enzyme (30 min) and stained for 10 min with 3′,3-diaminobenzidine tetrahydrochloride (DAB), counterstained with hematoxylin, dehydrated and mounted in Toluene-Free Mounting Medium (Dako). Positive control consisted of normal colon. Negative controls consisted of slides stained with IgG1κ murine melanoma immunoglobulin (Sigma-Aldrich, St. Louis MO; cat. # M9035) at the same concentration.

IHC scoring: Staining was scored by a surgical cytopathologist (MVdS), using a 0–4 scale as follows: 0 = no staining, 1 = 1–5%, 2 = 6–25%, 3 = 26–75%, 4 = 76–100% of tumor cells. Only membrane expression was scored as positive.

### Statistical analysis

Statistical analysis was performed applying the SPSS-PC package (Version 29). Probability of < 0.05 was considered statistically significant.

The Mann–Whitney U test was applied to comparative analyses of occludin protein expression by IHC in HGSC vs. mesothelioma, as well as between effusions and surgical specimens separately for HGSC and mesothelioma.

The Mann–Whitney U test or the Kruskal–Wallis H test was applied to analysis of the association between occludin protein expression by IHC and clinicopathologic parameters (for 2-tier or 3-tier analyses, respectively) for patients with HGSC effusions. For this analysis, clinicopathologic parameters were grouped as follows: age: ≤ 60 vs. > 60 years; effusion site: peritoneal vs. pleural; FIGO stage: III vs. IV; chemotherapy status: pre- vs. post-chemotherapy specimens; residual disease (RD) volume: 0 cm vs. ≤ 1 cm vs. > 1 cm; response to chemotherapy: complete response vs. partial response/stable disease/progressive disease. Kruskal–Wallis H test was additionally applied to analysis of the association between occludin and previously studied members of the claudin family.

For progression free survival (PFS), follow-up time was calculated from the date of last chemotherapy treatment until the date of relapse, date of death from any cause or end of follow-up. Primary chemoresistance was defined as disease progression within ≤ 6 months, based on radiologic evidence and/or increase in CA 125 values. For overall survival (OS), follow-up time was calculated from the date of diagnosis until date of death from any cause or end of follow-up, whichever occurred first.

Univariate survival analyses of PFS and OS were executed using the Kaplan–Meier method and log-rank test. In this analysis, occludin protein expression was grouped as high vs. low based on cut-off at 25%. For patients with surgical specimens from several anatomic sites, staining results in only one specimen were included.

## Results

**Fig. 1:**
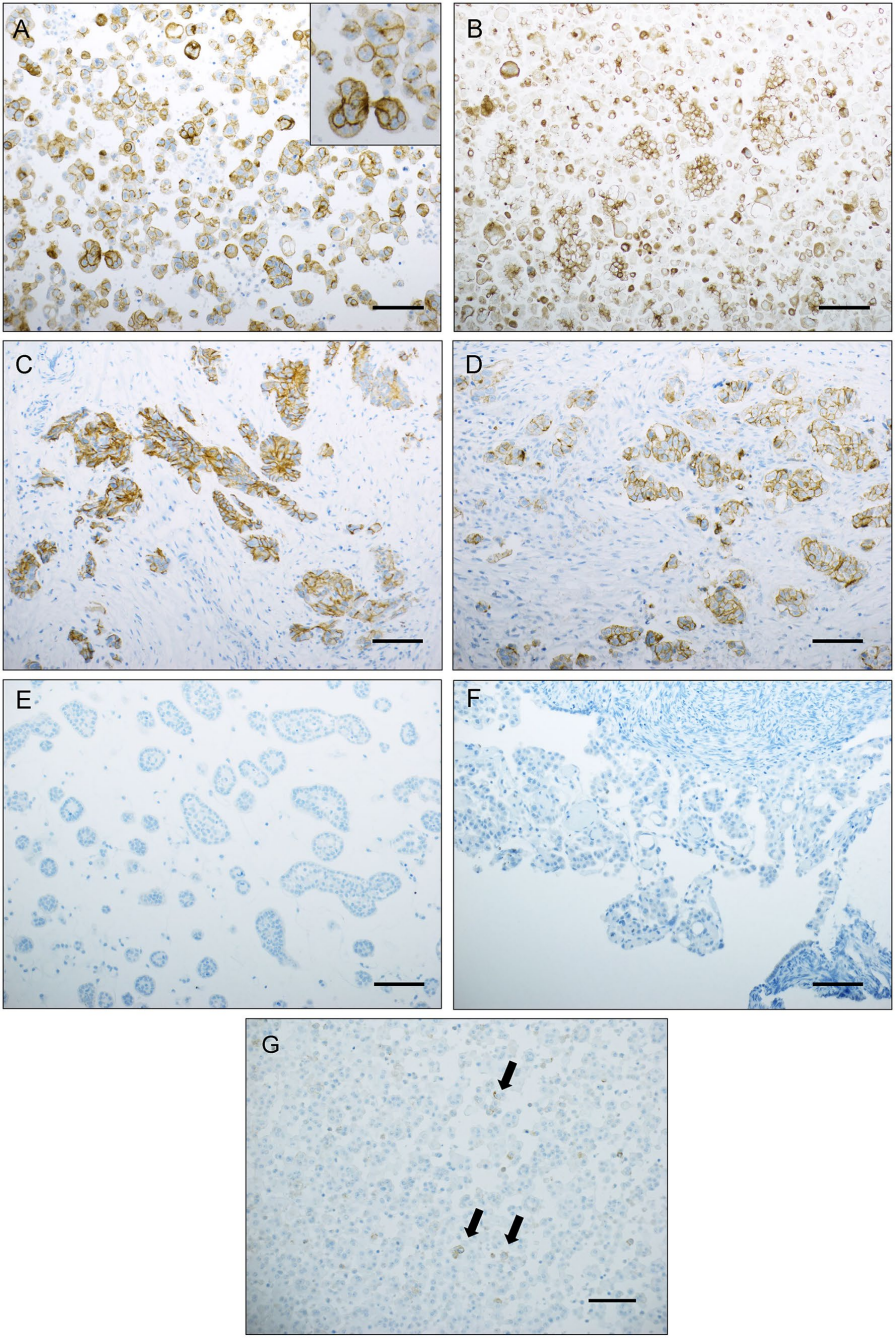
Immunohistochemistry. (**A–D**) Occludin protein expression in high-grade serous carcinoma (HGSC). (**A–B**) Effusion specimens; (**C–D**) Surgical specimens. High magnification inset in **A** highlights membrane staining. (**E–G**) Occludin staining in mesothelioma. Occludin-negative effusion and biopsy are shown in **E** and **F**, respectively. A mesothelioma effusion with focal expression (arrows) is shown in **G**. Scale = 100 µ in **A** and **C** to **F**, 200 µ in **B** and **G**.

Occludin protein expression was found in 587/602 (98%) HGSC vs. 40/87 (46%) mesotheliomas and was predominantly limited to < 5% of cells in the latter (Table 3**, **Fig. 1), with significantly higher expression in HGSC (p < 0.001). Occludin was additionally overexpressed in HGSC effusions compared to surgical specimens (p < 0.001). Expression in mesothelioma effusions and biopsies/surgical specimens did not differ (p = 0.902). Reactive mesothelial cells present in the HGSC effusions were negative for occludin.

**Table 3:** Occludin expression in HGSC and mesothelioma specimens

Specimen type and diagnosis	Staining extent (% of cells)					
	0%	1–5%	6–25%	26–75%	76–100%	p-value
HGSC effusions (n = 417)	10	30	64	174	139	p < 0.001^*a*^
HGSC surgical specimens (n = 185)	5	21	53	76	30	
Mesothelioma effusions (n = 45)	25	13	5	2	0	
Mesothelioma surgical specimens (n = 42)	22	17	3	0	0	

^*a*^For the comparison between HGSC and mesothelioma and between HGSC effusions and surgical specimens

Occludin was further overexpressed in post-chemotherapy HGSC effusions compared to chemo-naive effusions tapped at diagnosis (p = 0.015; Table 4). Expression was additionally higher in pleural compared to peritoneal HGSC effusions, though not significantly (p = 0.072). No association was seen with other clinicopathologic parameters in this patient group, including age (p = 0.776), FIGO stage (p = 0.394), RD volume (p = 0.161), CA 125 levels (p = 0.474), chemoresponse at diagnosis (p = 0.263) and primary resistance (p = 0.115).

**Table 4:** Occludin expression in chemo-naïve vs. post-chemotherapy HGSC effusions (n = 408 cases with known status)

Chemotherapy status	Staining extent (% of cells)					
	0%	1–5%	6–25%	26–75%	76–100%	p-value
Chemo-naïve (n = 272)	3	21	46	128	74	p = 0.015^*a*^
Post-chemotherapy (n = 136)	7	9	15	44	61	

^*a*^Significantly higher expression in post-chemotherapy effusions

In HGSC surgical specimens, occludin expression was associated with poor chemoresponse (p < 0.001) and primary resistance (p = 0.001, Table 5). No association was seen with other clinicopathologic parameters (p > 0.05; data not shown).

**Table 5:** The association between occludin expression in HGSC surgical specimens and chemotherapy response^*a*^

Chemotherapy response at diagnosis	Staining extent (% of cells)					
	0%	1–5%	6–25%	26–75%	76–100%	p-value
Complete (n = 80)	4	13	25	25	13	p < 0.001^*b*^
Partial (n = 27)	1	0	5	15	6	
Stable disease (n = 4)	0	0	0	2	2	
Progression (n = 15)	0	0	2	9	4	
Primary chemoresistance	Staining extent (% of cells)					
	0%	1–5%	6–25%	26–75%	76–100%	p-value
Yes (n = 46)	2	4	3	23	14	p = 0.001^*c*^
No (n = 97)	3	11	34	37	12	

^*a*^Data regarding chemoresponse at diagnosis and primary chemoresistance available for 126 and 143 specimens, respectively

^*b*^Higher expression in specimens from patients with poor (non-complete) response

^*c*^Higher expression in specimens from patients with primary chemoresistance

Survival data were available for 414 of the 417 patients with HGSC effusions, and the follow-up period ranged from 1 to 179 months (mean = 37 months, median = 31 months). PFS ranged from 0 to 148 months (mean = 11 months, median = 7 months). At the last follow-up, 370 patients were dead of disease, 23 were alive with disease and 10 were alive with no evidence of disease. Four patients died of complications and one patient died of unrelated causes. Six patients with initial follow-up were subsequently lost to follow-up.

Occludin expression in effusions was unrelated to survival in analysis of the entire cohort (p = 0.209), as well as in separate subgroup analysis of patients with chemo-naïve effusions and those with previous exposure to chemotherapy (p = 0.154 and p = 0.992, respectively). Similarly, no association with PFS was seen in analysis of the entire cohort (p = 0.726), patients with chemo-naïve effusions (p = 0.737) and patients with previous exposure to chemotherapy (p = 0.976).

Clinical parameters associated with OS in the entire cohort were patient age (p = 0.014), FIGO stage (p < 0.001) and RD volume (p = 0.003). FIGO stage (p = 0.003) and RD volume (p < 0.001) were also significantly associated with PFS. Survival curves for OS in the entire cohort are shown in Supplementary Fig. 1.

Clinical data were available for 91 of the 114 patients with surgical HGSC specimens. No association was observed between occludin expression and OS (p = 0.648) or PFS (p = 0.121; data not shown).

We recently studied the expression of claudin-10, another tight junction protein, in this cohort [6]. Statistical analysis showed significant association between expression of these proteins in 391 HGSC effusions immunostained for both (p < 0.001; mean rank = 161.95 vs. 207.23 for the low and high occludin expression group, respectively). Analysis of the association between occludin expression and expression of other claudin family members (claudin-1,-3,-4 and -7), which we studied some years ago [7, 8], did not show such association, though the number of specimens with both values was considerably smaller (n = 102; p > 0.05; data not shown).

## Discussion

Tumor cells in carcinomas have a dynamic balance between epithelial and mesenchymal characteristics, in which the relative expression of key molecules mediating epithelial differentiation and their negative regulators is continuously modified, the opposing processes termed epithelial-mesenchymal transition (EMT) and mesenchymal-epithelial transition (MET) [9]. We previously reported on changes in the expression of claudins [7, 8], E-cadherin and associated catenins [10], and regulators of EMT (Snail, Slug, SIP1) [11] in adnexal serous carcinomas along tumor progression. Significant association with patient survival was observed for several of these molecules. Given this, we were motivated to study occludin expression and its clinical role in metastatic HGSC.

No previous data are available with respect to the role of occludin in HGSC in the experimental or clinical setting. However, other groups have reported on the role of this molecule as mediator of cell migration and invasion in experimental models of lung carcinoma [12] and cancer-associated fibroblasts in colon carcinoma [13]. Occludin was further reported to promote angiogenesis experimentally in vivo in bladder cancer [14]. As reported for claudins, occludin may be over- or under-expressed during tumor progression, with evidence of the former in hepatocellular carcinoma [15] and the latter in endometrial carcinoma [16].

In the present study, occludin was significantly overexpressed in HGSC compared to mesothelioma specimens and was absent in reactive mesothelial cells, suggesting a potential diagnostic role. This finding is in full agreement with our previous reports with respect to claudins in these two malignancies [6, 7]. Whether occludin is differentially expressed in epithelioid and sarcomatoid mesothelioma cannot be resolved in the present study, as the vast majority of cases, including all effusions, had epithelioid differentiation. The fact that occludin is expressed in the majority of metastatic HGSC suggests that it may have other noncanonical functions other than maintaining tight junction structure and function in this cancer, potentially as regulator of migration, invasion and metastasis, as well as promotion of proliferation and suppression of apoptosis.

Occludin was additionally significantly overexpressed in HGSC effusions compared to solid metastases. Seen together with the overexpression of E-cadherin [10] and claudins [8] in effusions, a unique anatomic compartment, compared to solid tumors, HGSC cells are likely to be undergoing MET when present in the anoikis-resistant growth conditions of serous effusions, as previously reported by our group [17]. Of note, occludin and claudin-10 were significantly co-expressed in HGSC effusions, in staining of serial sections from the same cell block, despite the fact that the latter was under-expressed rather than over-expressed in effusions compared to surgical specimens [6], suggesting that their co-expression may provide survival advantage in the effusion microenvironment.

Occludin was further overexpressed in post-chemotherapy effusions compared to chemo-naive effusions. While these specimens were not paired, the higher occludin expression in post-chemotherapy effusions, the majority from patients with disease recurrence, suggests an association between expression of this molecule and acquired resistance to chemotherapy. This would also imply that higher occludin expression may be a marker of disease progression.

The hypothesis linking occludin to chemotherapy resistance is supported by another observation in the present study, i.e., the association between expression of this protein in surgical HGSC specimens and poor chemoresponse at diagnosis, a continuous variable, as well as intrinsic chemoresistance, a dichotomous variable with cut-off at 6 months. In our previous study of claudin-10, higher expression of this protein was found in surgical specimens obtained following neoadjuvant chemotherapy compared to chemo-naïve tumors [6]. Thus, the findings of both studies suggest that higher expression of tight junction proteins is related to tumor progression and aggressive disease in HGSC.

No significant association between occludin expression in effusions or solid specimens and disease outcome was found in the present study, despite the association with poor chemoresponse and intrinsic resistance in surgical specimens. This concurs with the findings of Nakanishi et al. in bladder carcinoma [18] and may suggest that other factors may be involved in modifying this interaction.

The present study has several limitations. These include its retrospective design, the fact that the majority of specimens were not patient-matched, and the absence of molecular data related to HRD status, which may have impacted chemotherapy response and outcome. Additionally, despite the fact that the majority of patients received platinum-based chemotherapy, differences related to dosage and drug combinations, as well as surgery extent, exist, as treatment for HGSC has evolved during this period. Finally, the molecular mechanism by which occludin may confer chemoresistance needs to be explored in vitro and in vivo. The present study nevertheless provides the first evidence suggesting a clinical role for this molecule in HGSC.

In conclusion, our study demonstrates that occludin is overexpressed in metastatic HGSC compared to mesothelioma and may represent a novel diagnostic marker of this tumor, particularly in effusion specimens. Occludin appears to be associated with disease progression in HGSC and may predict chemotherapy response in this cancer, though this should be validated in other series. Changes in the expression of tight junction proteins in effusions compared to solid metastases provide further support to our earlier observations of dynamic balance between EMT and MET as function of anchorage vs. growth in suspension of tumor cells.

### Supplementary Information

Below is the link to the electronic supplementary material.

Supplementary file1 (PPTX 5028 KB)—**A:** Kaplan–Meier survival curve showing the association between occludin protein expression in 411 HGSC effusions and overall survival (OS). Patients with effusions with high (>25%) occludin expression (n=309; red line) had mean OS of 40 months compared to 44 months for patients with effusions showing low (≤25%) expression (n=102, blue line; p=0.209). **B:** Kaplan–Meier survival curve showing the association between patient age and OS for 411 HGSC patients. Older (>60 years) patients (n=236; red line) had mean OS of 38 months compared to 45 months for younger (≤60 years) patients (n=175, blue line; p=0.014). **C:** Kaplan–Meier survival curve showing the association between FIGO stage and OS for 397 HGSC patients with advanced-stage disease. Patients diagnosed with stage IV disease (n=153; red line) had mean OS of 31 months compared to 46 months for patients with stage III disease (n=244, blue line; p<0.001). **D:** Kaplan–Meier survival curve showing the association between residual disease (RD) volume and OS for 327 patients with debulking data. Patients debulked to no macroscopic disease (n=72; blue line) had mean OS of 60 months compared to 44 and 39 months for patients debulked to 1 cm (n=136, red line) and ≥2 cm (n=119, green line), respectively (p=0.003)

## Data Availability

The datasets generated and analyzed during the current study are available from the corresponding author on reasonable request.
