# Supplementary figures and images for: Occludin is overexpressed in tubo-ovarian high-grade serous carcinoma compared to mesothelioma and is a marker of tumor progression and chemoresistance
Source: Clin Exp Metastasis. 2023 Dec 23;41(1):69–76. doi: 10.1007/s10585-023-10251-5 (PMC10830600; doi:10.1007/s10585-023-10251-5)

## Slide 1
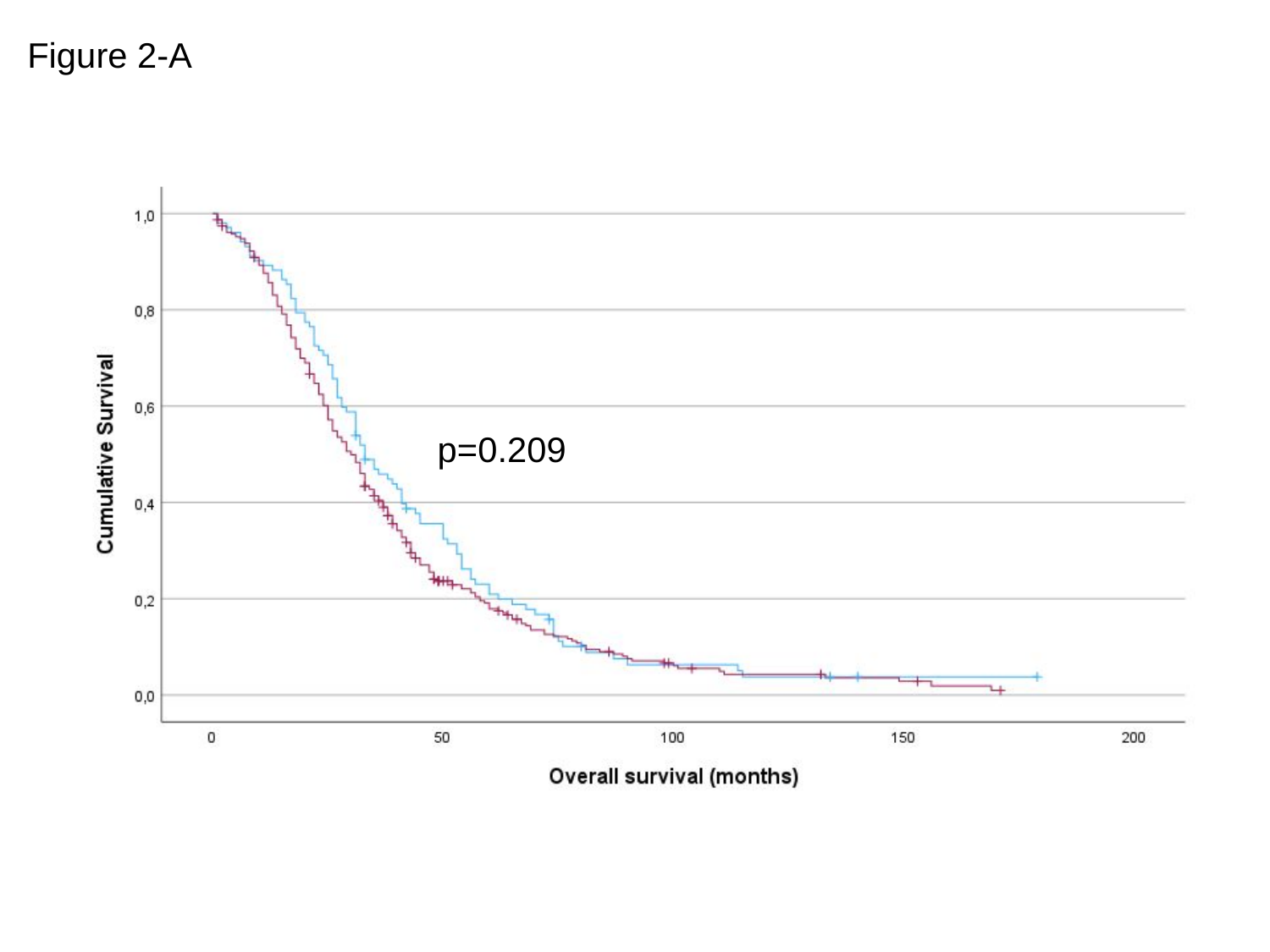

Figure 2-A
p=0.209

## Slide 2
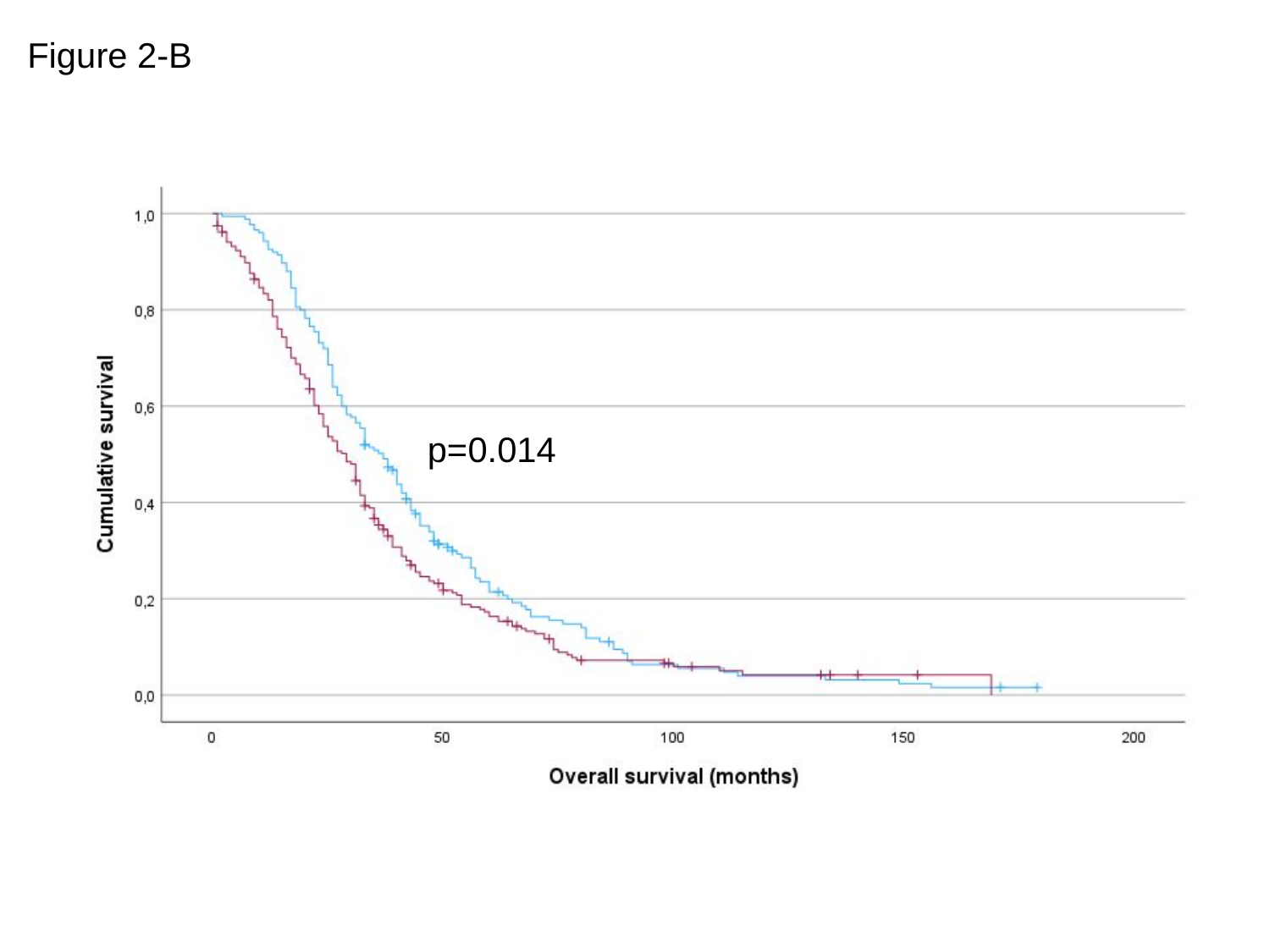

Figure 2-B
p=0.014

## Slide 3
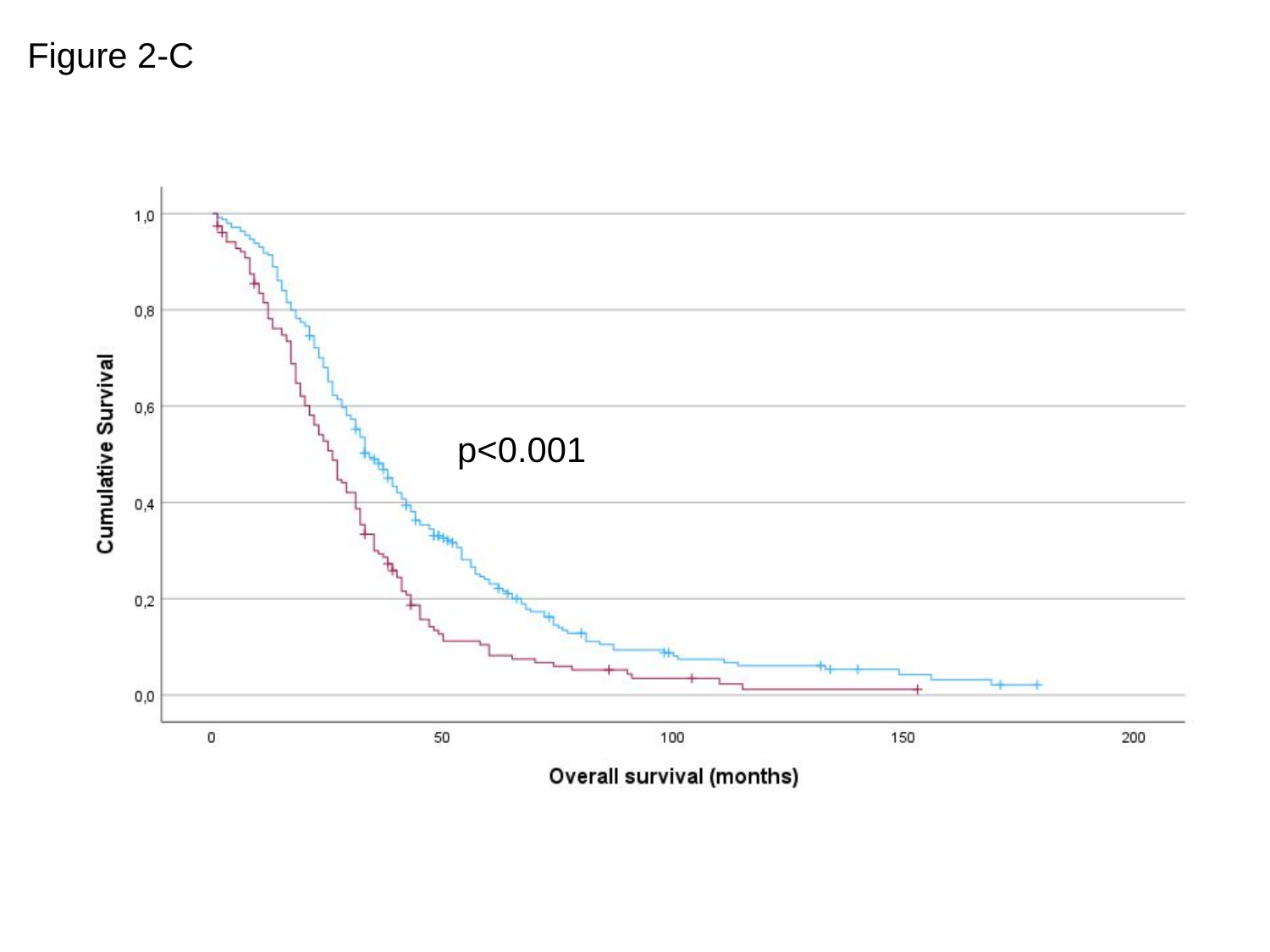

Figure 2-C
p<0.001

## Slide 4
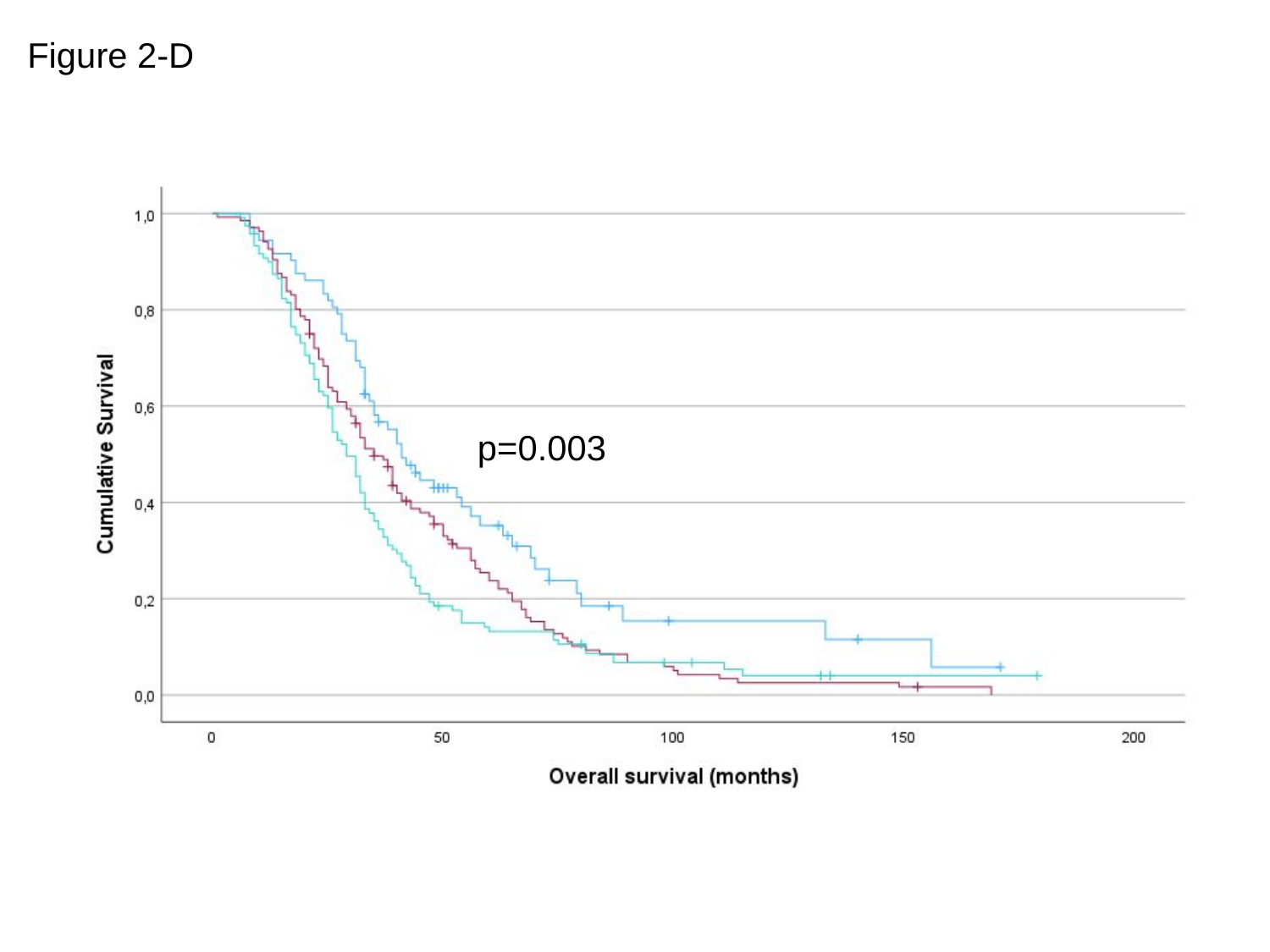

Figure 2-D
p=0.003

Supplement: Supplementary file 1 — Supplementary file1 (PPTX 5028 KB)—A: Kaplan–Meier survival curve showing the association between occludin protein expression in 411 HGSC effusions and overall survival (OS). Patients with effusions with high (>25%) occludin expression (n=309; red line) had mean OS of 40 months compared to 44 months for patients with effusions showing low (≤25%) expression (n=102, blue line; p=0.209). B: Kaplan–Meier survival curve showing the association between patient age and OS for 411 HGSC patients. Older (>60 years) patients (n=236; red line) had mean OS of 38 months compared to 45 months for younger (≤60 years) patients (n=175, blue line; p=0.014). C: Kaplan–Meier survival curve showing the association between FIGO stage and OS for 397 HGSC patients with advanced-stage disease. Patients diagnosed with stage IV disease (n=153; red line) had mean OS of 31 months compared to 46 months for patients with stage III disease (n=244, blue line; p<0.001). D: Kaplan–Meier survival curve showing the association between residual disease (RD) volume and OS for 327 patients with debulking data. Patients debulked to no macroscopic disease (n=72; blue line) had mean OS of 60 months compared to 44 and 39 months for patients debulked to 1 cm (n=136, red line) and ≥2 cm (n=119, green line), respectively (p=0.003) [file 10585_2023_10251_MOESM1_ESM.pptx]
